# The Role of the Airway and Gut Microbiome in the Development of Chronic Lung Disease of Prematurity

**DOI:** 10.3390/pathogens13060472

**Published:** 2024-06-04

**Authors:** Lieve Boel, David J. Gallacher, Julian R. Marchesi, Sailesh Kotecha

**Affiliations:** 1 Neonatal Unit, University Hospital of Wales, Cardiff CF14 4XW, UK; lieve.boel2@wales.nhs.uk (L.B.); david.j.gallacher@wales.nhs.uk (D.J.G.); 2 Division of Digestive Diseases, Faculty of Medicine, Imperial College, London W2 1NY, UK; j.marchesi@imperial.ac.uk; 3 Department of Child Health, Cardiff University School of Medicine, Cardiff CF14 4XN, UK

**Keywords:** preterm infant, chronic lung disease, bronchopulmonary dysplasia, gut microbiome, airway microbiome, probiotics

## Abstract

Chronic lung disease (CLD) of prematurity, a common cause of morbidity and mortality in preterm-born infants, has a multifactorial aetiology. This review summarizes the current evidence for the effect of the gut and airway microbiota on the development of CLD, highlighting the differences in the early colonisation patterns in preterm-born infants compared to term-born infants. Stool samples from preterm-born infants who develop CLD have less diversity than those who do not develop CLD. Pulmonary inflammation, which is a hallmark in the development of CLD, may potentially be influenced by gut bacteria. The respiratory microbiota is less abundant than the stool microbiota in preterm-born infants. There is a lack of clear evidence for the role of the respiratory microbiota in the development of CLD, with results from individual studies not replicated. A common finding is the presence of a single predominant bacterial genus in the lungs of preterm-born infants who develop CLD. Probiotic preparations have been proposed as a potential therapeutic strategy to modify the gut or lung microbiota with the aim of reducing rates of CLD but additional robust evidence is required before this treatment is introduced into routine clinical practice.

## 1. Introduction

Chronic lung disease (CLD) of prematurity, also known as bronchopulmonary dysplasia (BPD), is the most common morbidity in infants born extremely (<28 weeks’ gestation) or very (28–32 weeks’ gestation) preterm [1,2,3,4,5,6]. The reported incidence of CLD shows huge variation (10–89%) depending on the definition used, the group of infants studied and geographical location. However, despite advances in neonatal intensive care, the incidence of CLD has remained largely unchanged. This lack of improvement is generally attributed to the increased survival of the most extremely preterm-born infants and the lack of any specific targeted treatment to prevent the development of CLD [1,3,5,7].

Amongst the many ante-, peri- and post-natal risk factors known to be associated with the development of CLD, gestational age and birthweight are associated with the highest rates of CLD, especially in those born at the lowest gestational ages and with the lowest birthweight, reflecting intrauterine growth restriction. Several other risk factors for the development of CLD have been described reflecting the complex and multifactorial aetiology of CLD [1,4,5,6,8].

The role of chorioamnionitis in the development of CLD remains controversial. Whilst prenatal inflammation stimulates lung maturation and, therefore, reduces the severity of neonatal respiratory distress syndrome, it potentially increases the risk of developing CLD by disturbing normal lung maturation and growth. As an important cause of preterm delivery, chorioamnionitis is at least an indirect risk factor for the development of CLD [1,6,9]. An association between colonisation of the respiratory tract with *Ureaplasma* spp. and subsequent development of CLD has been shown by several meta-analyses [10,11]; however, treatment of pulmonary *Ureaplasma* spp. was not associated with improvement in survival without development of CLD in the recently published AZTEC study [12].

Another important risk factor is mechanical ventilation, especially if administered invasively via an endotracheal tube, as it can cause barotrauma and/or volutrauma, resulting in inflammatory changes and subsequent alveolar damage. The use of supplemental oxygen leads to the production of reactive oxygen species, which have adverse effects on the developing lung. Therefore, both mechanical ventilation and supplemental oxygen are important risk factors in the development of CLD [1,6,7].

Observations have been made that postnatal systemic sepsis increases the odds of developing CLD. Equally, a decrease in the incidence of CLD has been noted when there is a reduction in neonatal sepsis. It is thought that sepsis causes lung inflammation directly via direct stimulation of lung cells by microbial ligands and indirectly due to a rise in systemic pro-inflammatory cytokines. The latter is also thought to be the reason for the increased incidence of CLD in preterm infants with necrotising enterocolitis [4].

**Figure 1:**
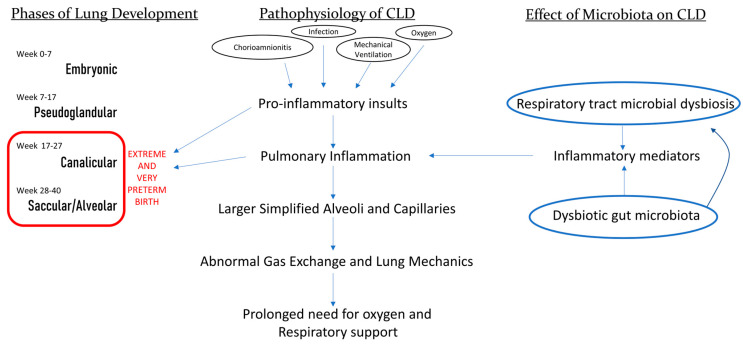
Mechanism of development of CLD in preterm-born infants and the potential effect of the microbiota on the pathophysiology of CLD.

The lungs of infants born before 32 weeks’ gestation are in the canalicular (17–27 weeks’ gestation) or the saccular (28–36 weeks’ gestation) stages of lung development. Exposure of the immature and developing lung to insults, such as invasive mechanical ventilation and supplemental oxygen, results in pulmonary inflammation which interferes with normal alveolarisation and microvascular development. The resulting changes in larger simplified alveoli and capillaries lead to abnormal gas exchange and lung mechanics, resulting in the CLD phenotype, characterised by a prolonged need for supplemental oxygen and/or respiratory support [1,3,4,5,6,7,8]. Figure 1 demonstrates the stages of lung development, the suggested pathophysiology of CLD and the potential effects of the lung and gut microbiota in influencing lung disease processes and lung growth. Definitions of CLD vary and are described in detail elsewhere [2,13,14].

Clinically, CLD not only results in prolonged neonatal hospital stay, it also predisposes to significant long-term morbidity. Infants with CLD have higher rates of respiratory tract infections and hospital readmissions than term-born and preterm-born infants without CLD. The structural lung changes, seen on CT scans, include hyperlucent areas, linear and triangular subpleural opacities and functional abnormalities with airflow obstruction and air trapping persisting into adulthood. It is now generally accepted that subjects who had CLD in infancy are at increased risk of developing early-onset chronic obstructive pulmonary disease (COPD) due to their failure to achieve optimal lung function at their peak in early adult life. Other than its respiratory consequences, CLD also predisposes to adverse neurodevelopment and cardiorespiratory health [1,5,7].

Despite advances in neonatal care, there are limited treatment options to prevent the development of CLD. Prevention of preterm birth is the ultimate solution, but to date, this aim has not been possible. Therefore, various other strategies, attempting to reduce rates of CLD, have been introduced into clinical neonatal practice. Corticosteroids administered antenatally to mothers at risk of preterm delivery have resulted in a reduction in neonatal respiratory distress syndrome and neonatal mortality, but they have not affected the incidence of CLD, possibly as a result of the more immature surviving at the expense of developing CLD. Non-invasive ventilation from birth and less invasive surfactant administration have been shown to potentially reduce the incidence of CLD. When mechanical ventilation is unavoidable, the use of gentler ventilation strategies with volume-targeted ventilation and permissive hypercapnia are recommended. Administration of excessive supraphysiological oxygen concentrations should be avoided, both during resuscitation at birth and in the neonatal intensive care unit, but using lower oxygen saturation targets (85–89%) is associated with increased mortality when compared to higher saturation limits (91–95%). Postnatal corticosteroids, administered systemically or via inhalation, do reduce CLD, but are not without important side effects, especially of the neurological system and therefore, their use varies widely. Caffeine, a methylxanthine derivative used to treat apnoea of prematurity, secondarily also reduces rates of CLD. Infants with CLD have high caloric requirements because of their increased work of breathing, lung healing and lung growth, therefore they require optimal nutritional strategies, including adequate protein administration. Recently, exogenous stem cells or progenitor cells have been shown to protect or regenerate damaged lung tissue, but before such therapies can be introduced into clinical practice, further studies are needed to prove their efficacy and safety in randomised controlled trials [1,5,6,7,15].

Since the underlying mechanisms in the development of CLD remain poorly understood, any new scientific developments will need to be carefully evaluated. The airway and gut microbiota, and their possible interrelation and potential role in the development of CLD, are only just being addressed in preterm-born infants at risk of developing CLD, but their modulation can potentially provide alternative therapeutic avenues to ameliorate rates of CLD in this vulnerable population [8].

## 2. Gut Microbiota and CLD

The human gut is known to host the greatest number and highest density of microorganisms of any site within the human body. The gut microbiota has been implicated in having a role in many disease processes, many of which are remote from the gut. These include conditions as diverse as cardiovascular [16], psychiatric [17] and autoimmune diseases [18].

Early microbial colonisation of the gut has been studied in detail in term-born infants unexposed to antibiotics and who were breastfed, to determine patterns of colonisation in the healthy infant during optimal conditions. These studies have shown that early microbial colonisation with facultative anaerobes, as pioneer species, predominate over the first few days of life, with increasing diversity over time [19]. This pattern of bacterial acquisition is influenced by perinatal factors, including mode of delivery, method of feeding and volume and type of antibiotic usage [20]. The infant gut microbiota is characterised by instability with rapid turnover of organisms within the gut, often with low diversity [21]. It can take up to two years for the gut microbiome to become stable and achieve adult patterns of gut microbial colonisation.

Preterm-born infants experience a very different exposome compared to healthy term infants, due to high levels of antibiotic exposure, slower introduction of enteral feeding and nursing in incubators with exposure to staff in the neonatal intensive care environment. The preterm gut is immature and thus likely to be a habitat with different characteristics to the more mature term gut. It is, therefore, unsurprising that the preterm gut microbiological colonisation differs from that of term infants. Extremely preterm-born infants, those most at risk of CLD, typically have lower rates of early colonisation with Bacteroidetes and Actinobacteria, but a relatively higher abundance of Firmicutes and Proteobacteria, than term-born infants [22].

Respiratory diseases, including cystic fibrosis, COPD and asthma, have associations with changes in the gut microbiota thought to contribute to disease pathogenesis and severity of disease [23]. Potential mechanisms for such effects have been proposed, including the microbiota’s ability to affect systemic inflammatory responses, short-chain fatty acids and the many other products of bacterial metabolism influencing host physiology or gene expression, or the concept of a gut–lung axis, whereby the microbiota within the lung is influenced by that within the gut with an effect on the host disease. Each of these mechanisms has the potential to have a role in the pathogenesis of CLD.

Indirect evidence of a potential role for the microbiota in the development of CLD is demonstrated by the effect of prolonged antibiotic use in the first two weeks of life on the risk of developing CLD. A large study controlled for gestational age and CRIB-II score (a measure of how unwell the infant is at birth) demonstrated that each day of antibiotic use was associated with an increased risk of developing CLD with greater severity [24]. Both the gut and respiratory microbiota are affected by antibiotic exposure [25], with the potential that antibiotic-induced perturbations of intestinal or airway bacteria may contribute to the development of CLD.

A recent study by Zhang and colleagues attempted to characterise links between the gut microbiota and CLD by assessing systemic inflammatory profiles. Preterm-born infants who developed CLD were shown to have reduced alpha diversity measures in stool samples collected at both 3–7 days and 14–28 days of age when compared to the infants who did not develop CLD. At 14–28 days of age, organisms from the Proteobacteria phylum were more prevalent, but Firmicutes were less prevalent in those who developed CLD when compared to those who did not develop CLD. These differences were associated with changes in serum cytokine profiles at days 14–28 with proinflammatory cytokines interleukin (IL)-1b, IL-4, IL-6, IL-8 and TNF-α being significantly higher in the CLD group [26]. Many factors are known to affect systemic cytokine levels; thus, unsurprisingly, this observational study, as with many such studies focusing on CLD as an outcome, was complicated by the CLD group having a lower birthweight and much earlier gestational age, factors which themselves can affect these outcomes, thereby limiting the interpretation. Adjustments for these important early life factors are often not made due to the small population sizes of most such studies.

Ryan and co-workers investigated the potential effect of the gut microbiota in preterm-born infants on host inflammatory gene expression related to CLD. *Escherichia*/*Shigella*, *Klebsiella* and *Salmonella*, all from the Enterobacteriaceae family, were reported to be associated with the development of CLD in vaginally born infants. In contrast, reduced relative abundance of *Bifidobacterium* was associated with the development of CLD in infants delivered by caesarean section. Infants with CLD had increased expression of genes involved in red blood cell development and oxygen transport, whereas immune-related pathways were downregulated. Correlations between host gene expression and specific taxa in the stool microbiota were identified with plausible biological mechanisms that may affect development of CLD. *Lactobacillus* was associated with enriched interferon signalling. *Staphylococcus* was associated with MAP kinase activation and members of the bacilli class (*Firmicutes phylum*) were associated with enrichment of CD71^+^-associated genes. *Bifidobacterium* in stool was found to be associated with the downregulation of inflammatory gene expression in blood. These associations deserve further evaluation for potential host–microbiota interactions which may contribute to the development of CLD or the protection against CLD [27]. The study included over 250 samples from 50 infants making it the largest study investigating the association between the gut microbiota and development of CLD. The authors also attempted to correct for gestational age, sex and birth mode in their statistical analysis, reducing the effect of confounders which most neonatal microbiota studies are affected by. Interestingly, none of the relevant studies have identified any association between *Clostridia* colonisation within the gut and CLD outcomes.

An alternative strategy for identifying evidence of bacterial influence in the pathogenesis of CLD is the analysis of volatile organic compounds (VOC). These carbon-based chemicals are largely produced by the bacterial organisms colonising the gut. Berkhout and colleagues demonstrated a difference in the VOC signature at days 14, 21 and 28 days of life, but not at 7 days of age between infants who did and did not develop severe CLD [28]. Interestingly, this small study showed no difference in the composition of the microbiota between the two groups. This lack of difference may be due to the low numbers of infants in the study, or may indicate the role of host–microbiota interactions, which may be more important than purely the organisms present in the microbiota.

Most microbiome studies target bacterial DNA to identify the constituents of the bacterial microbiota. Understanding the role that other non-bacterial microorganisms also present in the gut may have in disease is at a much earlier stage of research. Willis and co-workers sought to identify the fungal component of the microbiome, or mycobiome, in stool samples from preterm infants. They demonstrated an increased diversity of the mycobiome in samples from infants who developed CLD, and these infants had a lower relative abundance of *Candida* and a higher relative abundance of rarer fungal taxa including *Mortierella* when compared to those who did not develop CLD [29].

Animal models, e.g., in rodents, permit manipulation of the microbiota in a way not possible in observational studies on human subjects. This insight into microbiota–host interactions under controlled laboratory conditions has limitations in terms of extrapolation to CLD pathogenesis in human infants, but may be useful in identifying causal relationships between gut or lung microbiota and the development of CLD. One study has identified that antibiotic-induced disruption of gut microbiota acquisition leads to greater severity of CLD with increased mortality and pulmonary fibrosis in a hyperoxic mouse model of CLD [30]. This study did not analyse the respiratory microbiota, which presumably was also distorted by the antibiotic treatment. The fact that germ-free mice (i.e., free of any microbiota) are protected from the effect of hyperoxia compared to normally colonised mice suggests a role of the microbiota in mediating hyperoxic lung damage [31]. One possible mechanism that could explain this effect is the early colonisation causing a proinflammatory response in the lung. This is supported by the finding of lower concentrations of proinflammatory cytokines in the bronchoalveolar lavage fluid from germ-free mice compared to normally colonised mice [31]. A proinflammatory or infective effect of early colonisation has also been suggested in human studies [25].

The association between the gut microbiome and the development of CLD may be more complex than the impact of the gut bacterial composition on the pathogenesis of CLD. Hyperoxia, a well-recognised risk factor for developing CLD, is associated with changes in the gut and lung microbiota in experimental animal models [32,33]. The interaction of hyperoxia affecting the microbiome and conversely the microbiome mediating the effect of hyperoxic lung injury demonstrates the complexity of the biological systems contributing to the development of CLD. Despite the research in both human studies and animal models, a reproducible and modifiable association between gut colonisation and developing CLD is yet to be found.

## 3. Respiratory Microbiota and CLD

Our team has previously investigated the gut–lung axis, demonstrating that these two niches harbour different populations of microorganisms in extremely preterm-born infants at risk of CLD [25]. This was despite the likely common sources of early colonisation from the maternal and environmental microbiota.

Early colonisation of the respiratory tract is affected by similar exposures to that of the gut including the use of antibiotics during the early neonatal period and mode of delivery. Similar primary colonising genera are found within the respiratory tract as in the gut, which is unsurprising considering the limited exposures of such young infants to sources of colonising bacteria. Infants born by caesarean section tend to have a greater relative abundance of staphylococcal organisms while vaginally delivered infants have increased Gram-negative organisms including *Acinetobacter* and *Serratia* species [25].

Over recent years, several studies have investigated the role of organisms colonising the respiratory tract during the development of CLD. Significant variations exist in the anatomical niche sampled to represent the lung microbiome. Most studies focus on the more easily sampled upper respiratory tract (nasal/oropharyngeal) microbiota, using this as a proxy for lung colonisation. However, one study has demonstrated a difference in the microbiota of the upper and lower respiratory tract highlighting the weakness of this assumption [25]. Infants requiring mechanical ventilation can have tracheal samples obtained via an endotracheal tube from the upper airways or the lower airways can be sampled by using bronchoalveolar lavage. These samples are more likely to more accurately capture the colonisation within the lungs, but run the risk of contamination due to contact with the nasopharynx or with the endotracheal tube during sampling.

Several studies have detected bacterial DNA in amniotic fluid and the placenta during pregnancy suggesting that the respiratory tract may be exposed to bacteria well before birth [34,35]. Despite a robust rebuttal of these papers, which concluded that the detected microbial DNA was more likely to come from contamination of samples than genuine bacterial colonisation [36], the possible colonisation of the foetus remains a controversial subject. One recent paper analysing bacterial DNA found in amniotic fluid taken during preterm labour unsurprisingly found a greater bacteria load in these samples compared to amniotic fluid taken from pregnancies with intact membranes when labour was not threatened [37]. As infection is a risk factor for preterm birth, infants exposed to chorioamnionitis or subject to prelabour rupture of membranes may begin colonisation before birth. The same study examined the association of the development of CLD with bacterial DNA found in amniotic fluid samples collected just before birth. A higher rate of the *Escherichia*/*Shigella* cluster in infants with moderate/severe CLD was noted, while less severely affected infants had more *Ureaplasma* spp. and *Enterococcus* present [37]. Several studies have demonstrated very low yields of bacterial DNA detection from respiratory samples taken in the first few days of life [25], indicating that any colonisation of the respiratory tract before birth is likely to be of extremely low biomass and that established and stable communities of organisms are unlikely to present in the airways during foetal life. However, another study examining samples from the upper respiratory tract taken within 24 h of birth identified bacterial DNA in all 25 samples, showing conflicting results [38].

Understanding the possible role of the respiratory microbiota in the pathogenesis of CLD has been a source of interest to many researchers since the technology of next-generation sequencing has become available to analyse the low biomass of microbiological communities typically found in the airways and lungs. The bacterial load of the upper respiratory tract respiratory mucosa is a fraction of that found in the gut, but its proximity to the lower airways may enable colonising organisms to influence the physiological and pathological processes contributing to, or protecting against, the development of CLD. There is a plausible biological mechanism through which the respiratory microbiota could contribute to the development of CLD. The presence of pathogenic bacteria in the lower airways results in an infective process [39] and is likely to result in pulmonary inflammation which is strongly associated with the development of CLD. Alternatively, the preterm-born infant’s immune system could actively respond to the lower level of bacterial biomass associated with early colonisation, also resulting in pulmonary inflammation [25].

Differences in bacterial diversity are frequently used in microbiome studies to indicate an “unhealthy” microbiome. A small study of 25 infants published in 2014 demonstrated that infants who developed CLD had a lower total number of species and lower Shannon diversity index than infants who did not develop CLD [38]. These results were not replicated in a larger study of 152 infants which also used tracheal aspirate samples, taken on day 7 of life [40]. These variable results could be due to differing patient populations, local staff and environment, or due to sampling or sequencing methodologies used.

Specific taxa have also been implicated in some studies in contributing to the development of CLD. Organisms from the *Firmicutes phylum* increased and from the *Proteobacterium phylum* decreased over time in those infants who subsequently developed CLD [38]. This change in phyla was contradicted by a later study which reported that a decrease in Firmicutes and an increase in Proteobacteria over time may be associated with development of CLD [41]. A further study identified a difference in *Staphylococcus* and *Ureaplasma* spp. colonisation in the first few days of life, with infants who developed CLD having relatively less *Staphylococcus* and relatively more *Ureaplasma* spp. colonisation than infants who did not develop CLD [40]. Before the introduction of next-generation sequencing techniques, *Ureaplasma* spp. infection was consistently identified as a potential risk factor for developing CLD [10]. The relative abundance of *Ureaplasma* spp. identified in tracheal aspirate samples in one study showed it was often a highly dominant organism, suggesting this may be an infective process rather than a colonisation process. This study also highlighted a difference in the turnover of organisms in the airways of infants who developed severe CLD [40].

A more recent small study sampled the nares of preterm-born infants of <30 weeks’ gestation at birth at 1 and 3 weeks of age and identified an increased relative abundance of *Prevotella* and decreased relative abundance of *Caulobacter* in the CLD group at both time points [42].

**Figure 2:**
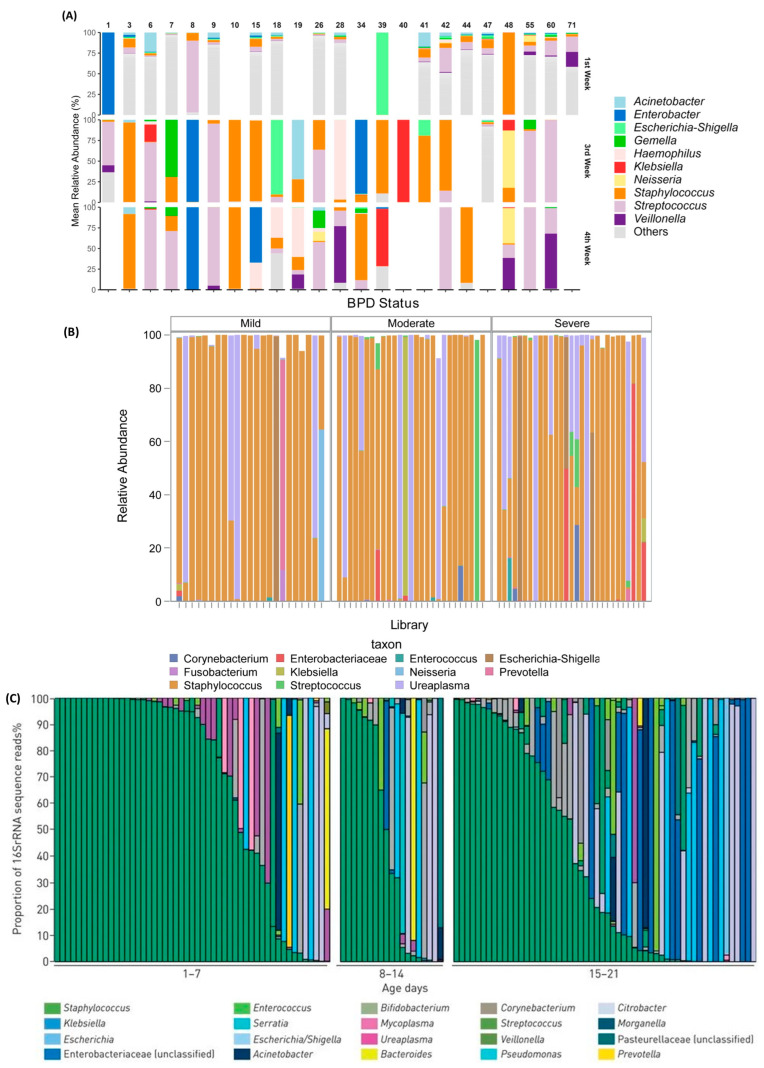
Panel figure demonstrating presence of a dominant genus (>50% of reads in a sample) in the majority of samples from different anatomical sites within the respiratory tract of preterm-born infants, repeated across multiple studies. Each bar in each figure represents one clinical sample from a preterm infant. (**A**)—Saliva samples [47], (**B**)—tracheal aspirate samples [40], (**C**)—nasopharyngeal aspirate samples [25].

The published literature, therefore, highlights the inconsistencies and lack of clear evidence for a role of the respiratory microbiota in the development of CLD. A consistent finding between almost all the studies has been the presence of a single bacterial genus dominating the early respiratory microbiota (>50% of sequencing reads in a sample) in preterm infants [25,38,40,43]. Figure 2 shows results from three studies sampling different respiratory sites, showing the presence of a dominant bacterial genus despite sampling from different respiratory tract sites of preterm-born infants. The other consistent finding has been the individual patterns of colonisation and turnover of organisms in the respiratory tract of preterm-born infants [25,40]. This ecology may reflect the diverse exposures and multiple contributing factors to the colonisation by bacteria of the airways, which may explain the different patterns of colonisation which contribute to the development of CLD. A 2019 systematic review [44] concluded that there was no conclusive evidence that the respiratory microbiota influenced the pathogenesis of CLD, however, this may be due to predominant organisms being infective rather than passive members of the stable microbiome.

Table 1 summarises the studies that have used 16S rRNA gene sequencing to identify the respiratory microbiota of preterm infants and identify a role for the microbiota in the pathogenesis of CLD. This table highlights the different methodological approaches taken including the differences in sample taking methodology, different regions of the 16S rRNA gene sequenced and the differences in inclusion criteria. There are also differences in the DNA extraction methodology between the studies, different geographical locations and very different success rates in amplification of the bacterial DNA. Most of the studies have utilised relatively small populations of infants. Each of these factors may have contributed to the difference in the results between studies. All of the studies listed in Table 1 utilised a robust and well-documented methodology. Each of them showed considerable overlap of the common phyla and genera identified as contributing to the bacterial microbiome, organisms expected to be found in the human host. This suggests a degree of repeatability in the core findings between the studies and is reassuring when considering the possibility of contamination, known to affect low biomass microbiota studies. All the studies in Table 1 are observational studies and subject to the potential confounding such methodology introduces. The risk factors for developing CLD, including the degree of prematurity, in-utero growth restriction and chorioamnionitis may also affect early bacterial colonisation. Postnatal management including postnatal corticosteroid and antibiotic use can not only affect development of CLD but also bacterial colonisation. Overall, most, but not all, studies focussing on CLD have reported the relevant confounding variables to permit interpretation of their effect on bacterial colonisation. However, only Wagner et al. [40] have included potential confounding variables in their statistical models to look at the effect of ventilation days and steroids on the lung microbiome.

Significant evidence suggests that early colonisation of the lungs is associated with a proinflammatory pulmonary response in the host. Peaks of neutrophil proteinases in bronchoalveolar lavage fluid from preterm infants are associated with the presence of a predominant organism in ventilated preterm infants [45] and peaks of the proinflammatory cytokines IL-6 and IL-8 in bronchoalveolar lavage fluid have been associated with a detectable microbiota in another study [25]. High levels of the same cytokines were also associated with detectable microbiota in a further study, suggesting that the respiratory microbiota acquisition may promote proinflammatory immunological reactions and contribute to the inflammatory response that ultimately leads to development of CLD.

Probiotics are treatments to prevent colonisation by pathogens and are given to many preterm infants usually with the aim of reducing the risk of necrotising enterocolitis (NEC). A meta-analysis of randomised controlled trials, which focussed on whether oral probiotics prevented the development of NEC in preterm-born infants, also assessed if the probiotic treatment decreased the rates of CLD. No effect was noted on rates of CLD after oral probiotics treatment in infants born at <32 weeks’ gestation [46]. However, the included studies were variable using different probiotic preparations, with preparations containing either a single strain or multi-strains, thus the results need to be interpreted with caution.

## 4. Potential Mechanisms of Microbiota Effect on CLD

Each infant appears to have a unique bacterial colonisation pattern from birth and this microbiome is different between anatomical sites within the body [25]. When the airway and gut microbiome are examined early in infants who subsequently develop CLD, it has been shown that the microbiota have less bacterial diversity, greater microbial community turnover and over time show changes in the relative abundance of the dominant bacterial phyla [8,26,38,40,41,44,48] than those of infants who do not develop CLD. To date, it is unclear if airway microbial composition is a causal factor or if it increases the infant’s susceptibility to the development of CLD [8,48].

Suggested mechanisms by which the composition of the airway and gut microbiota may contribute to CLD development include immunomodulation, oxidative stress and metabolic dysregulation [8,48]. Specific respiratory tract microbes can result in the release of pro-inflammatory cytokines and T helper 17 (Th17) cell activation. Th17 cells secrete cytokines of the interleukin-17 family, which modulate the local mucosal barrier function and, as with other pro-inflammatory cytokines, activate pulmonary fibrosis. Neutrophil migration is induced when lung microbiota alters the function of alveolar macrophages, dendritic cells, invariant natural killer T-cells, T-regulatory cells or lung resident gamma delta T-cells. Inflammatory mediators in immature lungs can restrict the activity of surfactant proteins and vascular endothelial growth factors, which could explain the alveolar and vascular changes seen in CLD [48]. Oxidative stress amplifies the inflammatory process by activating the NLRP3/caspase-1 pathway, which promotes interleukin-1b production [48]. Microbial metabolites, short-chain fatty acids or tryptophan catabolites can mediate inflammation [49]. High concentrations of endotoxins, such as lipopolysaccharides, have been detected in the airways of infants who develop CLD. This component of the outer membrane of Gram-negative bacteria activates airway epithelial cells, neutrophils and alveolar macrophages, resulting in the release of inflammatory mediators [41,48,49]. The gut–lung axis, the influence of the lung microbiome by the gut microbiome, is another potential factor in CLD development. In any gut microbiome, potential pathological bacteria can predominate, causing reduced microbial diversity and an increased inflammatory response, secondarily activating the lung immune response. When dendritic cells recognize pathogens in the intestinal tract, they present the antigens to T-lymphocytes in the mesenteric lymph nodes, activating T-cell subsets which produce cytokines and migrate to the respiratory tract, activating an inflammatory response in the lungs [26,48,50].

## 5. Future Research Direction

This review has demonstrated that the role of the microbiota on lung health in preterm-born infants is not well understood. Research into CLD should remain a priority for the neonatal research community in view of the ongoing significant clinical burden of CLD and the lack of effective treatment [12]. Within this research environment, understanding the effect of bacterial colonisation on pulmonary inflammation should be prioritised. Further work should analyse pulmonary inflammatory profiles and identify associations with colonisation patterns or with specific dominant organisms. As inflammation is thought to be the common pathway through which risk factors for CLD affect disease progression, preventing or minimising any pro-inflammatory responses occurring in response to early bacterial lung colonisation should be prioritised. Probiotics may have a role to play in modifying the inflammatory response; however, better understanding of the mechanisms of host–bacteria interaction are required before instituting studies of probiotics to modulate the lung or stool microbiome to prevent development of CLD.

Most existing studies of the respiratory microbiota have focused on ventilated infants, due to the ability to sample the lower airways. Neonatal practice is changing with a trend towards managing extreme preterm-born infants on non-invasive respiratory support due to concerns over ventilator-associated lung injury [51]. Unfortunately, sampling of the lower airways is not possible from a non-ventilated infant so establishing the development of the “normal” respiratory microbiome is not possible, especially as the nasopharyngeal airways poorly reflect the lower airways in this population. Innovative techniques are required to sample the lower airways which could include, for instance, sampling of the lower airways during non-pulmonary surgical operations e.g., for inguinal hernia repair. Until such methods are available and routinely applied, most studies will continue to focus on those requiring invasive mechanical respiratory support, thus representing the extremes of lung disease.

## 6. Conclusions

The gut and airways of preterm-born infants have similar early colonising bacterial taxa. These infants at risk of CLD have a range of different early life exposures compared to healthy term-born infants with the capacity to influence early bacterial colonisation. Individual studies have shown differences in the gut and respiratory microbiota, such as reduced bacterial diversity and greater turnover of organisms, in infants developing CLD compared to those without CLD. Therefore, a potential role of the microbiome in the development of CLD has been suggested. Potential mechanisms by which microbiota can contribute to the development of CLD are immunomodulation, oxidative stress and metabolic dysregulation. Inconsistencies between studies demonstrate that the potential role of the microbiota in the development of CLD remains uncertain. This requires further elucidation before probiotics or other strategies to attempt to reduce rates of CLD in extreme preterm-born infants via modification of the microbiome are introduced into clinical practice.

## Figures and Tables

**Table 1:** Appraisal of published literature on the airway microbiota in infants at risk of CLD, including studies using sequencing of the 16S rRNA gene sequencing to establish microbiota composition.

Study	Study Design	Geographical Location	Inclusion Criteria	Number of Preterm Infants	Number of Respiratory Samples Collected	Number of Respiratory Samples with Successful 16S rRNA Gene Sequencing	Region of 16S Gene Sequenced
Mourani et al., 2011 [43]	Prospective observational study using serial tracheal aspirate samples	USA—single centre	Infants born ≤34 weeks gestation and birth weight 500–1250 g requiring ventilation for >21 days	10	40	32	V1–V2
Lohman et al., 2014 [38]	Prospective observational study using serial tracheal aspirate samples	USA—single centre	Infants born ≤32 weeks gestation intubated within first 24 h of life	25	41	39	V3–V5
Lal et al., 2016 [41]	Prospective observational study using single tracheal aspirate sample collected after birth in discovery and validation cohort. Five infants underwent serial sampling. 18 infants with established CLD also included	USA—2 centres	Infants born ≤34 weeks gestation or birth weight 500–1250 g	55	69	69	V4
Wagner et al., 2017 [40]	Prospective observational study with a cross-sectional and longitudinal cohort study	USA—2 centres	Infants born ≤34 weeks gestation and birth weight 500–1250 g	201 (152 with suitable samples)	294 *	294 *	V1–V2
Gallacher et al., 2020 [25]	Prospective observational study using serial sampling of bronchoalveolar lavage, tracheal aspirate and NPA samples. No non-CLD comparison group	UK—2 centres	Infants born ≤32 weeks gestation requiring mechanical ventilation	55	904	207	V3–V4
Xu et al., 2022 [42]	Prospective observational study using nasal swabs collected at 1 and 3 weeks of age	China—single centre	Infants born ≤30 weeks gestation	28	52	49	V3–V4

* Inferred from results in paper.
